# Low-Arsenic Accumulating Cabbage Possesses Higher Root Activities against Oxidative Stress of Arsenic

**DOI:** 10.3390/plants12081699

**Published:** 2023-04-19

**Authors:** Hanhao Li, Yongtao Li, Xing Li, Xun Wen Chen, Aoyu Chen, Li Wu, Ming Hung Wong, Hui Li

**Affiliations:** 1 School of Environmental Science and Engineering, Shaanxi University of Science & Technology, Xi’an 710021, China; 2 Guangdong Provincial Research Center for Environment Pollution Control and Remediation Materials, College of Life Science and Technology, Jinan University, Guangzhou 510632, China; 3 College of Natural Resources and Environment, South China Agricultural University, Guangzhou 510642, China

**Keywords:** food safety, hydroponics, metalloid, root, soil contamination

## Abstract

Cabbage grown in contaminated soils can accumulate high levels of arsenic (As) in the edible parts, posing serious health risks. The efficiency of As uptake varies drastically among cabbage cultivars, but the underlying mechanisms are not clear. We screened out low (HY, Hangyun 49) and high As accumulating cultivars (GD, Guangdongyizhihua) to comparatively study whether the As accumulation is associated with variations in root physiological properties. Root biomass and length, reactive oxygen species (ROS), protein content, root activity, and ultrastructure of root cells of cabbage under different levels of As stress (0 (control), 1, 5, or 15 mg L^−1^) were measured As results, at low concentration (1 mg L^−1^), compared to GD, HY reduced As uptake and ROS content, and increased shoot biomass. At a high concentration (15 mg L^−1^), the thickened root cell wall and higher protein content in HY reduced arsenic damage to root cell structure and increased shoot biomass compared to GD. In conclusion, our results highlight that higher protein content, higher root activity, and thickened root cell walls result in lower As accumulation properties of HY compared to GD.

## 1. Introduction

Arsenic (As) has been identified as a carcinogen by health organizations, and excessive intake will affect human health [1]. The survey results show that As pollution is increasing in global agroecosystems [2,3]. Arsenic content in paddy soil could be eight times higher than the background value [4]. Vegetables grown in contaminated soils could take up and accumulate As in their edible parts and elevate dietary As exposure to humans and animals [5,6]. The ability of crops to take up As is cultivar-dependent [7], and selecting low As-accumulating cultivars is an effective way to mitigate the problem [8,9].

Chinese flowering cabbage (*Brassica parachinensis* L.) is widely cultivated in Asia and is an essential component of the human diet [10]. The As-accumulating ability of cabbage is highly cultivar-dependent. For example, one cultivar could accumulate a low level of As concentration in the roots, which was approximately 15-fold lower than other cultivars [11]. One of the reducing mechanisms is involved with the higher As fixation in the root cell wall of the low-As accumulating cultivar, and the As translocation from root to shoot is limited [12]. These studies focused on the process of As transfer from roots to shoots but paid less attention to the process from outside media (e.g., soil pore water/solution) to roots. It is also not clear how the As stress affects the root physiological properties, which can essentially influence the As uptake and translocation.

The plant root is the first affected organ under soil environmental stress and is essential for reducing pollutant uptake and maintaining normal crop growth [13]. Arsenic root interactions may involve the following three aspects: (1) Contaminants affect root performance [14], such as decreasing root biomass and root length [15]; in turn, the altered root morphological characteristics and physiological fitness affect the uptake and transport of contaminants [16]. According to Kubo et al. [15], the root morphology (such as biomass, length, and the number of root tips) predominated the As uptake and subsequently influenced plant growth. The enhanced lignification/suberization of root endodermal cells under stress assists in plant stress tolerance and decreases the translocation of contaminants from roots to shoots [17]. (2) Roots are critical organs that balance oxidative stress and antioxidant activities under stress and contribute to plant stress tolerance [18,19]. Oxidative stress induced by reactive oxygen species (ROS) is generated in plant tissues when excessive As accumulation occurs [20]. Excessive production of ROS further damages the structure and properties of phosphate bilayers and biological macromolecules, which causes cell death, reduced water and nutrient absorption, and growth suppression [21]. (3) Several physiological changes in root tissues and cells serve as intrinsic factors for the uptake and bioaccumulation of pollutants by plants [13]. For example, Armendariz et al. [22] disclosed that overaccumulation of As was caused by a reduction in the surface area of the cortex and damaged cells in the outer layer. Zhao et al. [23] observed a better ultrastructure of mitochondria, endoplasmic reticulum, and nucleus in the root tip of cabbage accompanied by low Cr accumulation. The above findings suggest that root physiological parameters affect plant tolerance. However, the detailed information about whether As-induced alterations in these physiological processes are related to As accumulation in cabbage remains unclear and thus requires further study.

To reveal the roles of root physiological parameters in contributing cabbage As tolerance and accumulation, we used two cultivars with contrasting growth performance and As-accumulating abilities for comparison. The objectives of this study were (1) to investigate the changes in physiological indicators of root systems exposed to different As concentrations by comparing the two cultivars, and (2) to reveal the eventual association of root physiological indicators with cabbage As tolerance and accumulation. We hypothesized that reduced root arsenic uptake and reactive oxygen species accumulation in the low-accumulation cultivar promote increased shoot biomass. Increased root protein concentration and structural stability of root cells play a key role in resisting high levels of arsenic stress. 

## 2. Results

### 2.1. Biomass and Root Length

**Figure 1:**
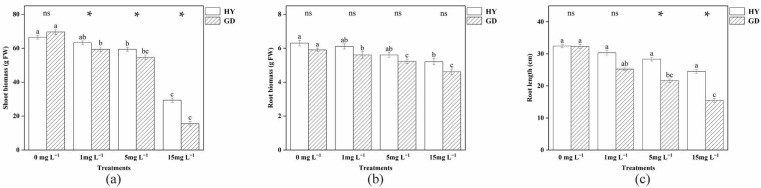
Shoot biomass (**a**), root biomass (**b**), and root length (**c**) of two Chinese flowering cabbages under different doses of As by ANOVA test. * indicates significant (*p* < 0.05) differences between two cultivars at the same As treatment by *t*-test. Different letters indicate significant (*p* < 0.05) differences among different As treatments in the same cultivar, the same below.

The shoot biomass of both cultivars decreased as As concentration increased. With increasing As concentration, HY was higher than GD, with the maximum difference being 89.03% at 15 mg L^−1^. In HY, 1 and 5 mg L^−1^ As exposure had no significant (*p* > 0.05) impact on the root biomass, and further increasing As level decreased this value (Figure 1a). In 1 and 5 mg L^−1^ As treatments, the root biomass of GD was lower than that of the control, but the difference in root biomass of HY was not significant (Figure 1b). At As stress, the root length of HY and GD progressively declined, with a difference between the two cultivars in 5 and 15 mg L^−1^ As treatments (Figure 1c). Upon the increasing As concentration, the root length of GD gradually decreased (*p* < 0.05). In contrast, the root length of HY decreased slightly (*p* > 0.05).

### 2.2. Arsenic Concentration and Speciation

Table 1 depicts the As concentration in the Chinese flowing cabbages. The shoot As content HY was reduced by 29.22% and 38.99% compared to GD at As concentrations of 1 and 5, respectively. Among them, As (III) was reduced by 26.62% and 33.71%, respectively. As (V) was reduced by 25.58% at an As concentration of 1 mg/l, the difference was insignificant at the arsenic concentration of 5 mg/L. There was no significant difference in all speciation of arsenic in both cultivars at an arsenic concentration of 15 mg/l. Under As treatment, root As concentration increased progressively (*p* < 0.05) from 13.45 to 177.56 mg kg^−1^ DW in HY, and from 25.93 to 238.35 mg kg^−1^ DW in GD, respectively. In the 1 and 15 mg L^−1^ As treatments, the total, organic (MMA and MDA), and inorganic (As (III) and As (V)) arsenic content in HY were lower than those in GD. In the 5 mg L^−1^ As treatment, there was no significant difference in total arsenic, but the As (III) and MMA contents in HY were higher than those in GD. The DMA and As (V) contents were lower.

### 2.3. Accumulation of O_2_^−^, H_2_O_2_ and MDA Content

**Figure 2:**
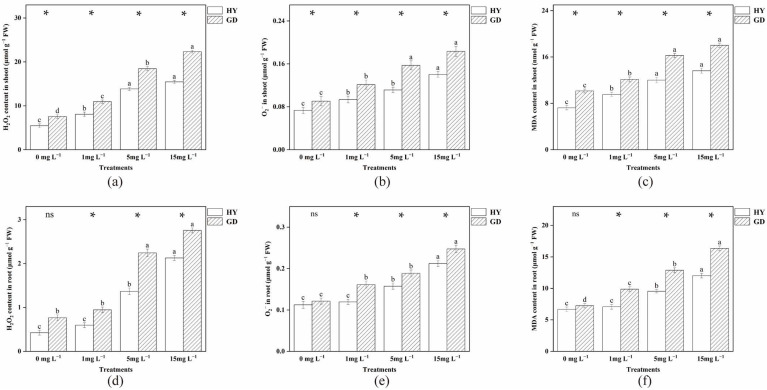
Shoot H_2_O_2_ (**a**), O_2_ (**b**), MDA content (**c**) and root H_2_O_2_ (**d**), O_2_^−^ (**e**), and MDA content (**f**) of two Chinese flowering cabbage under different doses of As. * indicates significant (*p* < 0.05) differences between two cultivars at the same As treatment by *t*-test.

Generally, the H_2_O_2_, O_2_^−^, and MDA content of the selected cultivars were enhanced with the increased As concentration (Figure 2). In the shoot, different concentrations of arsenic treatment all resulted in higher H_2_O_2_, O_2_^−^ and MDA content in the GD treatment than in the HY treatment. In the root, compared with the control treatment, 1 mg L^−1^ As addition had no significant difference in H_2_O_2_ and O_2_^−^ contents in HY, but O_2_^−^ content in GD was increased. H_2_O_2_ and O_2_^−^ contents were increased in both cultivars at arsenic concentrations of 5 and 15 mg L−^1^. Moreover, at the same As treatment, both H_2_O_2_ and O_2_^−^ concentrations in root were higher in GD than that in HY, with a significant difference observed between the two cultivars. Under the same concentration of arsenic stress, the MDA content of HY roots was lower than that of GD.

### 2.4. Analysis of Root Protein Content, Root Activity, and Glutathione (GSH)

**Figure 3:**
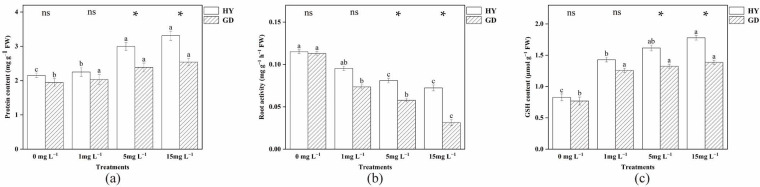
Root protein content (**a**), root activity (**b**), and GSH content (**c**) of two Chinese flowering cabbage under different doses of As. * indicates significant (*p* < 0.05) differences between two cultivars at the same As treatment by *t*-test.

Figure 3 depicts how As stress affected the protein content of *B. parachinensis* L roots. Compared to the control, 5 and 15 mg L^−1^ increased root protein content in HY. Yet for GD, the addition of arsenic had no significant effect. HY root protein content was higher at arsenic concentrations of 5 and 15 mg L^−1^ than in the GD. Root activity showed similar changes to root protein content (Figure 3b). Compared with the control treatment, 1 mg L^−1^ As addition markedly decreased the root activity of GD, but not HY. With the concentration of As increased from 1 mg L^−1^ to 15 mg L^−1^, the root activity of the two cultivars was reduced. It is also noted that the value of HY was higher than that in GD under 5 and 15 mg L^−1^ As treatments, respectively. Arsenic concentrations of 5 and 15 mg L^−1^ increased GSH activity in HY and were higher than in the GD treatment at the same concentration of arsenic stress.

### 2.5. Ultrastructure of Root Cells

**Figure 4:**
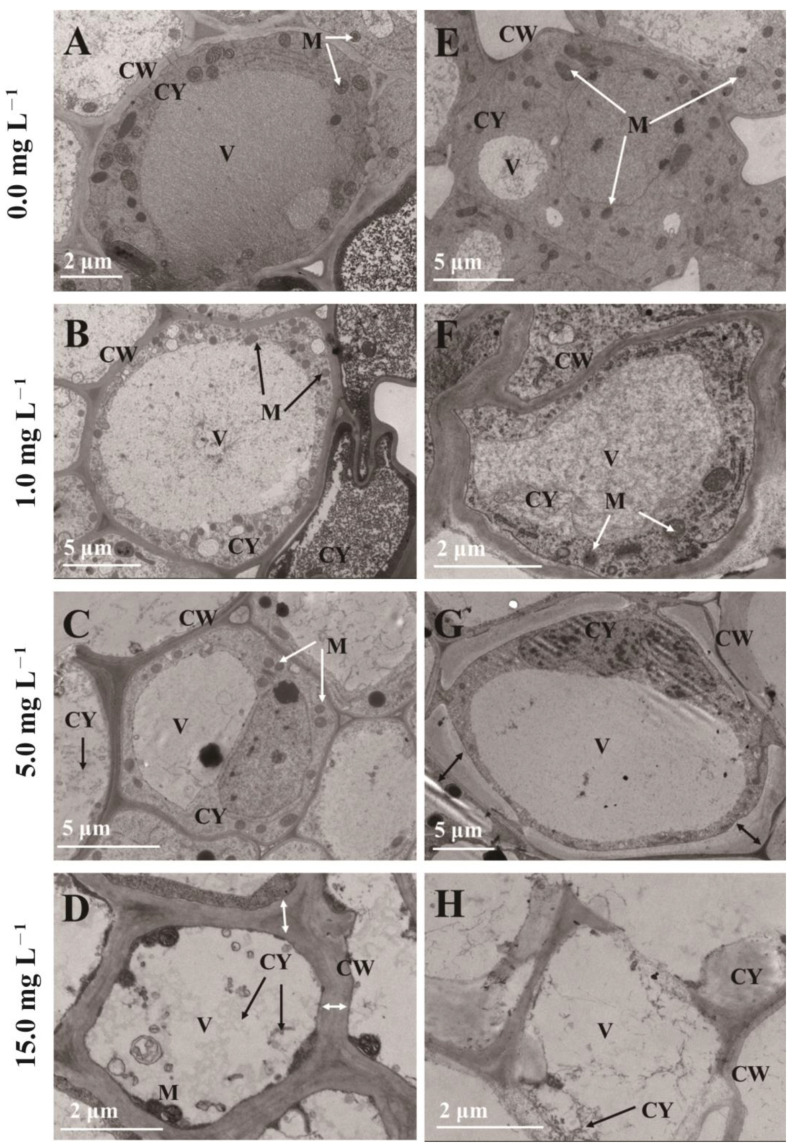
Transmission electron microscopy images of the ultrastructure of root tip cells under different treatments. HY (**A**–**D**), GD (**E**–**H**). CY: cytoplasm, CW: cell wall, M: mitochondria, V: vacuole.

TEM demonstrates the changes in the ultrastructure of root cells of two cultivars with increasing As concentration. Figure 4A,E presented the well structure and typical cellular ultrastructure of root cells under control conditions, with clearly delineated cell walls, plentiful mitochondria, and multiple organelles in the cytoplasm. For the 1 mg L^−^^1^ As treatment, the configuration of HY root cells was standard (Figure 4B), but a distortion of the cell wall and contraction of the cytoplasm were observed in GD. There was a partial separation of the cell wall and cell membrane and a relative decrease in mitochondria (Figure 4F). At an As concentration of 5 mg L^−^^1^, cell wall distortion and cytoplasmic vacuolization began to be observed in HY (Figure 4C). At the same time, vesicles in GD and also cytoplasmic boundaries began to blur and more severe plasmolysis could be observed (Figure 4G). The further As stress increased, the cytoplasm was gradually disorganized, and mitochondria gradually disappeared in HY and GD (Figure 4C,D,G,H). Moreover, the 15 mg L^−^^1^ As stress induced a thicker cell wall in HY (Figure 4D). We observed more severe damage on GD, such as cell wall breakage and organelle lysis, which implies that the root cell structure of GD has been disrupted (Figure 4H).

### 2.6. Correlation Analysis

**Figure 5:**
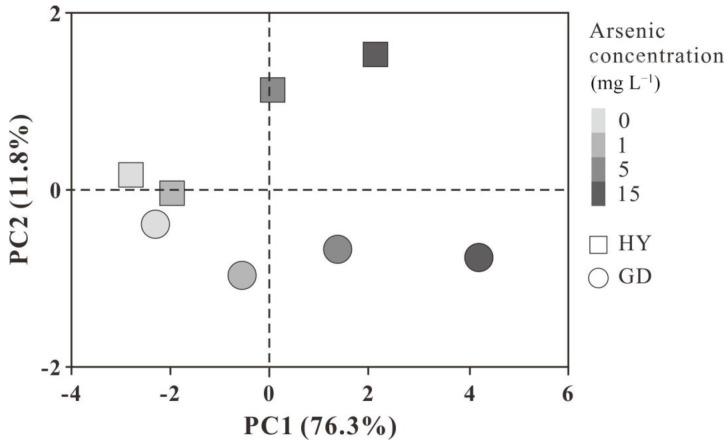
Principal component analysis (PCA) of plant growth parameters under different As concentrations.

The correlations between root biomass, length, As concentration, and physiological parameters in the two cultivars were investigated using Pearson correlation analysis. (Table 2). Results showed that root biomass and root length were and negatively correlated to As, H_2_O_2_, O_2_^−^, MDA, GSH contents in the root, and were and positively correlated to root activity, respectively. Root activity had a strong negative relationship with root As concentration. The first two axes could explain 88.1% of the total variance, according to principal component analysis (PCA), with PC1 and PC2 explaining 76.3% and 11.8% of the total variance, respectively (Figure 5). As stress tended to be distinguished by PC1, while cultivar differences were separated by PC2. The distances between control and As treatments in HY were closer than that of GD. Upon the increasing As level, the distance between HY and GD progressively increased.

## 3. Discussion

The primary organ for absorbing water and nutrients from the solution is the root, which is also the first section exposed to As. Hence, this function may be inhibited due to the overaccumulation of As in the root [4]. In the current study, 1 mg L^−1^ As treatments had reduced GD shoot biomass but had no significant effect on HY (Figure 1a). This was primarily due to differences in arsenic uptake in the two cabbage cultivars. Compared to HY, the As(III) and total As content of GD in the shoot was increased by 36.28% and 29.22%; in the root by 106.74% and 92.79% (Table 1). Many studies pointed out that exposure to contaminants is connected with the generation of ROS in plants [24]. ROS can affect the normal function of DNA and proteins by disrupting base and sugar structures [25]. As the second messenger, ROS can affect crop growth by altering the activity of superoxide dismutase and peroxidase [26]. A direct result of As-induced cellular changes is excessive ROS accumulation, which consequently causes oxidative stress to biomolecules [27]. Increased arsenic concentrations in the root induced higher levels of ROS (Figure 2). These results are reflected visually in the root cell structure. Under the 1 mg L^−1^ As treatment, HY displayed a normal cell structure (Figure 4B), but cytoplasm shrinkage and the reduction of mitochondria were observed in GD. As the “powerhouse”, mitochondria provide energy for root cell activity. The decrease in mitochondria was also following the decreased root activity, suggesting that GD was more sensitive to As stress (Figure 4F), and thus more easily suffered damage, in comparison to HY. This is consistent with the findings of Farooq et al. [28], who also reported that high exposure concentration of As decreased the growth of four different cultivars of *Brassica napus*. The decrease in biomass is partially linked to the detrimental impact of As on crop cellular processes, where energy is needed to create stress-related chemicals, including phytochelatins and antioxidants [29].

The differences in growth parameters between the two cultivars gradually increased with increasing arsenic concentrations (Figure 5). As the arsenic concentration increased to 15 mg L^−1^, the root length of both cultivars decreased gradually (Figure 1c). Previous studies on wheat and cabbage have also found that arsenic can inhibit root growth [28,30]. Reduced mitotic activity of root meristems further slowed cell division in root tip meristems and prevented the elongation of newly produced cells [31]. With 5 and 15 mg L^−1^ arsenic exposure, shoot biomass was higher in HY than in GD, with no difference in root biomass, but root length was higher in HY compared to GD. Root physiological parameters, such as root elongation, root surface area, and root tip numbers, might be related to the difference in As uptake in cabbages [32,33]. In this study, the As concentration of 15 mg L^−1^ was lower by 10.31% and 25.50% in the aboveground and root systems of HY compared to GD, respectively. Pearson correlation analysis also showed a positive correlation between As concentration and ROS concentration in the root, and a negative correlation between root length and ROS production (Table 2). These results suggested that As accumulation triggered excessive ROS formation in the root, causing lipid peroxidation (Figure 2f) [34], which may interfere with the physiological activities of roots, such as nutrient (nitrogen, phosphorus) and water transport and metabolism, injuring root cell division, and thus inhibiting root growth [35,36]. Other studies regarding As toxicity in plant roots also found that the generation of ROS by As stress was the critical factor resulting in the inhibition of root elongation [28]. This indicates that the dynamic balance between ROS production and scavenging was disrupted by the excessive accumulation of As, and the ability of GD to regulate external stress was gradually lost as the As concentration continued to increase [37]. GD was sensitive to As stress, with a high As concentration in the root, resulting in the inhibition of root growth. In contrast, HY exhibited high performance in resisting As stress, with well-developed growth and low As accumulation in the root (Table 3).

Soluble protein is an important macromolecule substance that increases plant resistance to stress by supplying metabolites and energy through a variety of metabolic pathways [38]. A recent study has demonstrated that soluble protein was related to metal tolerance in plants [39]. Soluble proteins can chelate with metals entering the root system, reducing the migration ability and toxicity of metals that might be an adapting mechanism in the plant. The present results showed that 5 and 15 mg L^−1^ arsenic treatments increased the root protein content in the HY treatment and were higher than in the GD (Figure 3a). Similar results were shown for changes in the GSH content of the root (Figure 3c). Glutathione is an important non-enzymatic antioxidant that can directly promote the decomposition of hydrogen peroxide [40], participate in redox signal transduction, activate various defense mechanisms, and improve cell tolerance [41]. The accumulation of more proteins in HY may involve a detoxification mechanism that protects biomolecules, allowing cells to store contaminants in vesicles and prevent damage to normal function, thereby enhancing root tolerance to arsenic [42]. Under either level of As exposure, the root activity of GD was sustainably lower than that of HY (Figure 3b), with a difference between HY and GD under 5 and 15 mg L^−1^ As treatments, indicating that the nutrient uptake ability of GD was lower than that of HY. This difference in root activity between HY and GD again confirmed that the physiological response of cabbages plays a critical role in mediating their growth and As uptake.

The ultrastructure of the root reflected a negative relationship between the ability to maintain the organelle integrity and As exposure concentration (Figure 4). Upon the increased As concentration to 15 mg L^−1^, the disintegration of cytoplasm, and reduction of mitochondria were observed in both HY and GD (Figure 4C,D,G,H), implying that senescence of roots might have occurred [43]. In addition, arsenic stress caused severe damage to the organelle structure of the root system of GD, such as plasmolysis (Figure 4G), and rupture of the cell wall (Figure 4H), as compared to what was seen in HY. This indicated that HY showed higher tolerance to As toxicity than GD did. It is noted that 15 mg L^-1^ As stress induced a thicker cell wall in HY (Figure 4D), whilst causing cell wall rupture in GD (Figure 4H). The thicker cell wall in HY may have slowed down the efficiency of arsenic aggregation, which may be a way for apical cells to protect their normal physiological functions [12]. The rupture of the cell wall in GD reflected this cultivar suffered severe As toxicity, in line with the high ROS content, low root activity, and inhibition of root growth (Figure 1, Figure 2 and Figure 3).

## 4. Materials and Methods

### 4.1. Experimental Design

The Chinese flowering cabbage (*B. parachinensis* L.) cultivars Hangyun 49 (HY, low As-accumulating) and Guangdongyizhihua (GD, high As-accumulating) were selected for the comparative study. After being sterilized for 15 min with 10% H_2_O_2_ (*v*/*v*), HY and GD seeds were rinsed ten times in sterile deionized water. The seeds were germinated on seeding plates, and the seedlings with four leaves were transferred to 5 L pots containing 20% Hoagland nutrient solution added with 0, 1, 5, and 15 mg L^−1^ (AsHNa_2_O_4_.7H_2_O). The dosage of arsenic in the experiment was set concerning extreme environments and published papers [44,45]. The pH was adjusted to 7.5 using HCl or NaOH. A total of six plants were cultivated in each pot. Each treatment was replicated three times, giving 24 pots in total. The nutrient solution for each pot was renewed every four days. A greenhouse was used to grow cabbages hydroponically at a temperature of 25 °C and a relative humidity of 70–90% under natural sunlight. The positions of the pots were organized using the randomized complete block design.

### 4.2. Harvesting and Sampling

On day 36, plants were harvested and washed carefully with ultrapure water. Root and shoot samples were collected separately. After blotting with tissue paper, the fresh weight of the sample was weighed. From the bottom of the stem to the tip of the longest root, the root length was measured. Subsamples were lyophilized (LL3000, Thermos, Waltham, MA, USA) and stored at −80 °C for further testing.

### 4.3. Measurement of Arsenic Concentration

The freeze-dried samples (0.05 g) were digested with HNO_3_-HCL-HF (volume ratio of 1:3:1) by Microwave Digestion System (Milestone, BG, Italy), and the filtrate was analyzed by ICP-MS (Thermo Fisher Scientific, OH, USA) to determine As concentration. The same procedure used to generate the plants was used to prepare blanks and samples of the standard reference material (wheat, GBW07603, IGGE, Langfang, China) as a quality control measure. The check recovery was within 90–110%. Four forms (As(III), As(V), MMA, DMA) were separated using an inductively coupled plasma liquid chromatograph from Perkin Elmer Instruments Ltd. (AAS, PinAAcle 900T, Perkin Elmer, MA, USA) for the separation. Chromatographic column type: Dionex IonPac AS11-HC, size: 250 × 4, equilibration time: 20 min. Mobile phase composition: (A): 0.6 mL sulfuric acid, 0.3 mL nitric acid, and 3 mL ammonia (pH = 10); (B): ultrapure water. Mobile phase flow rate: 0.5 mL min^−^^1^. The chromatographic peaks of the samples are shown in the Supplementary Materials (Figure S1).

### 4.4. Analysis of Root Protein Content, GSH Content, and Root Activity

To determine the soluble protein content, 0.5 g of fresh samples were weighed, ground in liquid nitrogen, transferred to a centrifuge tube with 5 mL of ultrapure water, and centrifuged at 3000 r/min for 10 min. The supernatant was collected and brought to 10 mL (V (total). We then aspirated 0.1 mL of sample (V (extract)), added 5 mL of G-250 solution, mixed thoroughly, and measured the absorbance at 595 nm after two minutes. By using 100 μg/mL of bovine serum protein as a standard sample, the formula was calculated as follows [46]:

Protein content (mg/g) = (Protein content converted according to the marking line/ug × V(total))/ (M × V(extract)).

Fresh samples (0.5 g) were ground well in 3 mL of acidic extraction buffer (5% potassium phosphate and 1 mmol/L ethylenediaminetetraacetic acid, EDTA). The supernatant was collected for GSH content by centrifugation at 12,000× *g* for 20 min at 4 °C. [47].

Root activity, expressed as the reduction ability (mg g^−1^ h^−1^), was measured following the method described by Li et al. [48]. Add 5 mL of 0.4% (*w*/*v*) triphenyl tetrazolium chloride (TTC) and phosphate buffer (pH 7.5) to fresh root samples, respectively, and incubate for two hours in the dark. Then 2 mL of sulfuric acid and 4 mL of ethyl acetate were added, ground, and filtered, and supplemented with ethyl constant volume to 10 mL. The absorbance of the extract at 485 nm was recorded. Root activity was expressed as the TTC reduction (mg g^−1^ h^−1^) and calculated as:

Root activity = [TTC reduction mass (mg)]/ [fresh mass (mg) × time (h)]

### 4.5. Analysis of MDA Content and Reactive Oxygen Species (ROS)

A modified version of the procedure described by Chomkitichai et al. [49] was used to measure malondialdehyde (MDA). Sample (1 g) was ground with 10 mL of 5% trichloroacetic acid, shaken at 50 °C and 150 r min^−1^ for 30 min, 1 mL of sample was mixed with 1 mL of 0.67% thiobarbituric acid (TBA) and fixed to 5ml. In the next step, the mixture was boiled in water for 30 min, cooled for centrifugation, and the absorbance at 532 nm and 600 nm was measured to calculate MDA content (nmol g^−1^ FW).

For the H_2_O_2_ determination, 0.5 g of each plant sample was weighed, ground in liquid nitrogen, and placed in a centrifuge tube. Then 4.5 mL of 0.1 mol/l PBS buffer (pH = 7.4) was added and shaken. The tube was then centrifuged at 12,000 rpm for 15 min and the supernatant was collected. Then corresponding reagents in sequence according to the manufacturer’s instructions were added. H_2_O_2_ reference standard (163 mmol/L) and double-distilled water were included as positive and blank controls, respectively. The absorbance of each sample was measured at a wavelength (A) of 405 nm using a spectrophotometer (Analytik Jena, Thüringen, Germany). The H_2_O_2_ content was calculated as follows [50]:


H_2_O_2_ content (μmol/g FW) = [A(sample) − A(Control)]/[A(standard) − A(Control)] × 163 × 1000

To extract superoxide in plant samples, 0.5 g of each plant sample was weighed. The same procedure of extracting H_2_O_2_ was used to obtain the supernatant and to produce the complex with purplish-red color. Then corresponding reagents in sequence according to the manufacturer’s instructions were added. Vitamin C (0.15 mg/mL) and double-distilled water were used as positive and blank controls, respectively. The absorbance of each sample was then measured using the spectrophotometer at 550 nm (A). The capacity of producing superoxide per minute was calculated as follows [51]:


O_2_^−^ content (μmol/g FW) = [A(sample) − A(Control)]/[A(standard) − A(Control)] × 0.15 × 1000 (1)

### 4.6. Investigation of Root Ultrastructure

The ultrastructure of root cells was determined according to Li et al. [52]. The microstructure of the root sample was examined using transmission electron microscopy (Thermo Fisher, Talos F200S, OH, USA). The roots were fixed for 24 h in the solution mixing 2.5% glutaraldehyde before TEM analysis. The samples were cleaned with PBS three times, and then gradient drying was performed for minutes and repeated three times, and then gradient drying was performed with 30, 50, 70, 80, 90, and 100% ethanol. The root samples were centrifuged and embedded with epoxy resin, cut to 50 nm thickness by an ultra-thin sectioning machine, stained with lead citrate for 1–2 h, and placed on copper mesh for observation.

### 4.7. Statistical Analysis

Means and standard errors (SE) were used to express all data. Using SPSS 16.0, a one-way analysis of variance (ANOVA) and least significant difference (LSD) test were performed to determine significant differences between different As treatments within the same cultivar. The independent sample *t*-test was performed to determine significant differences in two cultivars at the same As treatment. Two-way analysis of variance (ANOVA) was used to determine the effect of cultivar and As concentration on the physical and chemical properties of the root. Variables including biomass, As concentration, root protein content, root activity, and ROS content were included in the principal component analysis (PCA) using R v3.6.2. Pearson correlation was conducted to establish relationships between different growth and physiological parameters. Figures were performed using Origin 2022.

## 5. Conclusions

The results of the present study revealed that the low- and high-As accumulating cultivars showed significant physiological differences in response to As stress. Compared with GD, the low-As accumulating cultivar HY has higher protein content, higher root activity, and organized root cell, and displayed a higher resistance to As stress. In contrast, GD suffered severe oxidative damage, resulting in a limitation in protein synthesis, a decrease in root activity, ruptures of cell walls, and subsequently influenced plant growth. Overall, our findings showed that the cultivar HY outperformed the GD in all areas where they were evaluated, suggesting that cultivar plays an important role in response to As stress. Cabbage has a higher arsenic bioaccumulation factor than other vegetables and can threaten human health throughout the food chain [53]. This study is important for revealing the physiological and ecological differences in arsenic tolerance of different cabbage genotypes, as well as for screening low-accumulation cabbage varieties suitable for safe production in arsenic-contaminated soil.

## Figures and Tables

**Table 1:** As speciation in two Chinese flowering cabbages under different doses of As.

Organ	As Concentration (mg L^−1^)	Treatments	As Speciation (mg kg^−1^)				Total As (mg kg^−1^)
			As(III)	MMA	DMA	As (V)	
Shoot	0	HY	0.18 ± 0.02 ^a^	0.14 ± 0.03 ^a^	0.027 ± 0.002 ^a^	0.074 ± 0.03 ^a^	0.50 ± 0.043 ^a^
		GD	0.18 ± 0.02 ^a^	0.15 ± 0.02 ^a^	0.023 ± 0.002 ^a^	0.072 ± 0.02 ^a^	0.50 ± 0.064 ^a^
	1	HY	2.26 ± 0.21 ^b^	1.70 ± 0.02 ^b^	0.29 ± 0.02 ^a^	0.86 ± 0.06 ^b^	5.51 ± 0.22 ^b^
		GD	3.08 ± 0.27 ^a^	2.50 ± 0.21 ^a^	0.34 ± 0.04 ^a^	1.08 ± 0.09 ^a^	7.12 ± 0.26 ^a^
	5	HY	4.45 ± 0.42 ^b^	2.61 ± 0.25 ^b^	0.70 ± 0.05 ^b^	1.21 ± 0.08 ^a^	9.31 ± 0.46 ^b^
		GD	5.95 ± 0.48 ^a^	3.34 ± 0.39 ^a^	0.87 ± 0.05 ^a^	1.30 ± 0.09 ^a^	12.94 ± 0.75 ^a^
	15	HY	7.34 ± 0.78 ^a^	2.83 ± 0.21 ^a^	1.48 ± 0.09 ^a^	1.28 ± 0.09 ^a^	13.31 ± 0.56 ^b^
		GD	7.67 ± 0.58 ^a^	2.81 ± 0.26 ^a^	1.51 ± 0.11 ^a^	1.32 ± 0.09 ^a^	14.84 ± 0.85 ^a^
Root	0	HY	0.88 ± 0.02 ^a^	0.63 ± 0.03 ^a^	0.19 ± 0.002 ^a^	0.35 ± 0.03 ^a^	2.41 ± 0.14 ^a^
		GD	0.92 ± 0.02 ^a^	0.72 ± 0.02 ^a^	0.17 ± 0.002 ^a^	0.37 ± 0.02 ^a^	2.58 ± 0.17 ^a^
	1	HY	5.49 ± 0.20 ^b^	4.14 ± 0.02 ^b^	0.71 ± 0.02 ^b^	2.12 ± 0.06 ^b^	13.45 ± 1.33 ^b^
		GD	11.35 ± 0.27 ^a^	9.10 ± 0.21 ^a^	1.04 ± 0.04 ^a^	4.03 ± 0.09 ^a^	25.93 ± 2.54 ^a^
	5	HY	33.15 ± 0.42 ^b^	9.58 ± 0.25 ^b^	5.21 ± 0.05 ^a^	9.1 ± 0.08 ^a^	70.27 ± 5.44 ^a^
		GD	35.71 ± 0.40 ^a^	19.22 ± 0.39 ^a^	3.50 ± 0.05 ^b^	7.38 ± 0.09 ^b^	68.82 ± 2.35 ^a^
	15	HY	97.81 ± 0.78 ^b^	36.14 ± 0.21 ^b^	19.21 ± 0.09 ^b^	16.85 ± 0.09 ^b^	177.56 ± 10.44 ^b^
		GD	128.2 ± 0.58 ^a^	46.31 ± 0.26 ^a^	24.37 ± 0.11 ^a^	19.71 ± 0.09 ^a^	238.35 ± 15.35 ^a^

Different letters indicate significant (*p* < 0.05) differences between two cultivars at the same as treatment by *t*-test.

**Table 2:** Pearson correlation coefficients between root biomass, length, As concentration and H_2_O_2_, O_2_^−^, protein, MDA, GSH contents in the root, and root activity.

	HY			GD		
	Root Biomass	Root Length	As Concentration in Root	Root Biomass	Root Length	As Concentration in Root
As concentration in root	−0.68 *	−0.62 *		−0.91 **	−0.8 **	
H_2_O_2_ in root	−0.64 *	−0.7 *	0.9 **	−0.84 **	−0.75 **	0.86 **
O_2_^−^ in root	−0.8 **	−0.66 *	0.98 **	−0.92 **	−0.9 **	0.92 **
Protein content	NS	NS	0.84 **	−0.63 *	−0.58 *	0.62 *
Root activity	0.86 **	0.67 *	−0.8 **	0.87 **	0.81 **	−0.84 **
MDA in root	−0.89 **	−0.80 **	0.98 **	−0.88 **	−0.91 **	0.93 **
GSH content	−0.87 **	−0.87 **	0.79 **	−0.76 **	−0.84 **	0.68 *

NS represents no significance. * Represents significance at the level of *p* < 0.05. ** Represents significance at the level of *p* < 0.01.

**Table 3:** Two-way ANOVA for the growth and physiological parameters.

	Root Biomass	Root Length	As Concentration	H_2_O_2_ in Root	O_2_^−^ in Root	Protein Content	Root Activity
Cultivar	*p* < 0.05	*p* < 0.05	*p* < 0.001	*p* < 0.001	*p* < 0.001	*p* < 0.01	*p* < 0.001
As concentration	*p* < 0.001	*p* < 0.001	*p* < 0.001	*p* < 0.001	*p* < 0.001	*p* < 0.001	*p* < 0.001
Cultivar × As concentration	NS	NS	*p* < 0.001	NS	*p* < 0.05	*p* < 0.05	*p* < 0.05

## Data Availability

The data that support the findings are presented in this paper. Other data are available from the corresponding author upon reasonable request.

## Supplementary Materials

The following supporting information can be downloaded at: https://www.mdpi.com/article/10.3390/plants12081699/s1, Text S1: The methods of determination and calculation of H_2_O_2_ and Superoxide [54], Figure S1: Chromatographic peaks of different arsenic species of the standard arsenic solutions, Figure S2: Chromatographic peaks of different arsenic species in a sample.
