# Supplementary material for: Low-Arsenic Accumulating Cabbage Possesses Higher Root Activities against Oxidative Stress of Arsenic
Source: Plants (Basel). 2023 Apr 19;12(8):1699. doi: 10.3390/plants12081699 (PMC10146792; doi:10.3390/plants12081699)
Supplement: Supplementary file 1 [file plants-12-01699-s001.zip › plants-2278060-supplementary.pdf]

Supplementary Material

Title: Low-Arsenic Accumulating Cabbage Possesses Higher Root Activities against Oxidative Stress of Arsenic

Hanhao Li [1], Yongtao Li [1,3], Xing Li [2], Xun Wen Chen [2], Aoyu Chen [2], Li Wu [2], Ming Hung Wong [2] and Hui Li [2,*]

[1] School of Environmental Science and Engineering, Shaanxi University of Science & Technology, Xi'an 710021, China

[2] Guangdong Provincial Research Center for Environment Pollution Control and Remediation Materials, College of Life Science and Technology, Jinan University, Guangzhou 510632, China

[3] College of Natural Resources and Environment, South China Agricultural University, Guangzhou 510642, China

* Correspondence: tlihui@jnu.edu.cn; Tel.: +86-2085223405

Supporting Information Includes:

Supporting Information Includes:

- 1 text
- 2 figures

Text S1: The methods of determination and calculation of H_2O_2 and Superoxide:

Hydrogen peroxide (H_2O_2)

The commercial kit (Hydrogen Peroxide assay kit, Nanjing Jiancheng Bioengineering Institute, Nanjing, China) was used to measure the H_2O_2 content. H_2O_2 can react with ammonium molybdate to form a stable yellow complex, which has a strong absorption peak at 405 nm, and its absorbance value is proportional to the concentration of hydrogen peroxide [1]. Based on this principle, 0.5 g of each plant sample was weighed, ground in liquid nitrogen, and placed in a centrifuge tube. Then, 4.5 ml of 0.1 mol/l PBS buffer (pH=7.4) was added and shaken. The tube was then centrifuged at 12000 rpm for 15 minutes, and the supernatant was collected. Then corresponding reagents in sequence according to the manufacturer's instructions were added. H_2O_2 reference standard (163 mmol/l) and double-distilled water were included as positive and blank controls, respectively. The absorbance of each sample was measured at a wavelength (A) of 405 nm using a spectrophotometer (Analytik Jena, Germany). The H_2O_2 content was calculated as follows:

H_2O_2 content (μmol/g FW) = [A(sample) - A(Control)]/ [A(standard) - A(Control)] * 163 * 1000

Superoxide (O_2^-)

Adding an electron-transferring substance and the Griess reagent to superoxide produces a complex with purplish-red color (550 nm). To extract superoxide in plant samples, 0.5 g of each plant sample was weighed. The same procedure of extracting H_2O_2 was used to obtain the supernatant. To produce the complex with purplish-red color. Then corresponding reagents in sequence according to the manufacturer's instructions were added. Vitamin C (0.15 mg/ml) and double-distilled water were used as positive and blank controls, respectively. The absorbance of each sample was then measured using the spectrophotometer at 550 nm (A). The capacity of producing superoxide per minute was calculated as follows:

O_2^- content (μmol/g FW) = [A(sample) - A(Control)]/ [A(standard) - A(Control)] * 0.15 * 1000

The chromatogram of the determination of 4 forms of As:

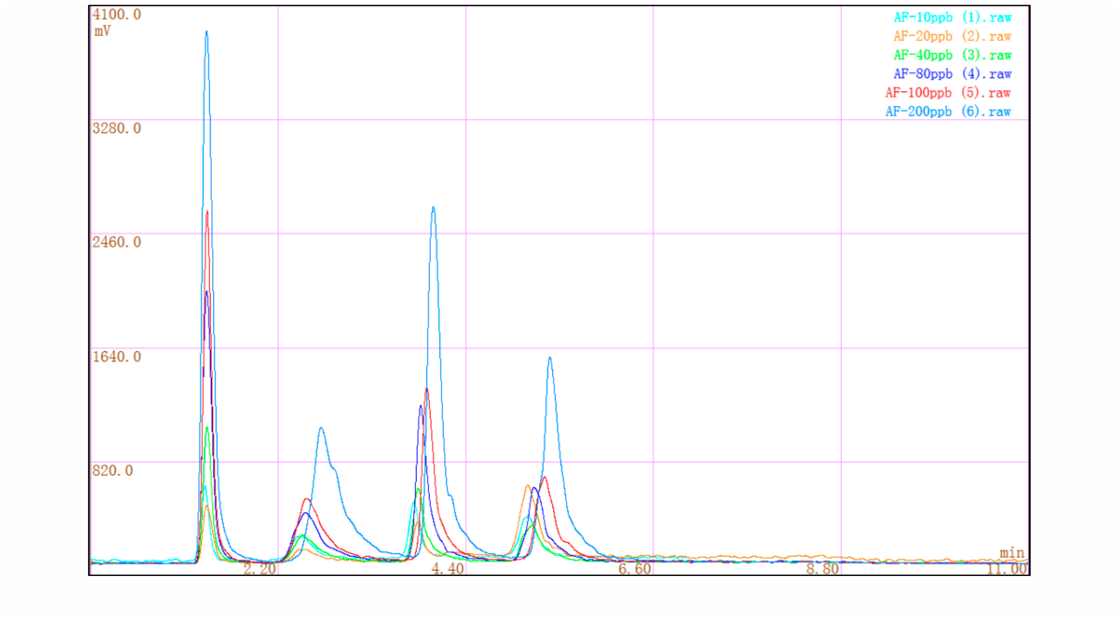

Figure S1. Chromatographic peaks of different arsenic species of the standard arsenic solutions.

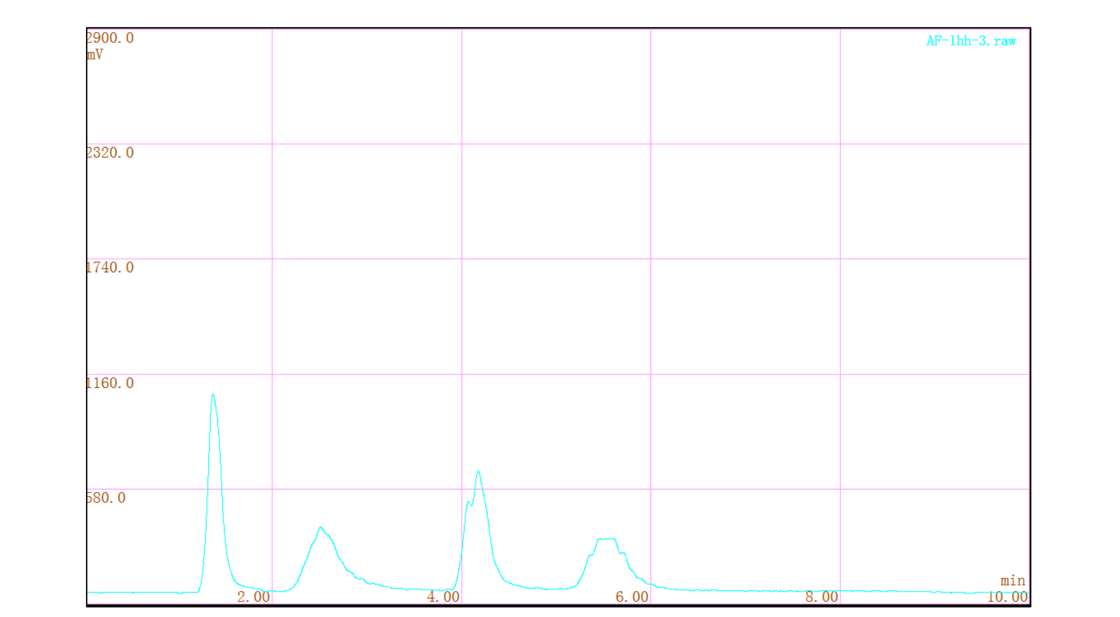

Figure S2. Chromatographic peaks of different arsenic species in a sample

Reference

1. Li, Y.; Zhang, Q.; Yu, Y.; Li, X.; Tan, H. Integrated proteomics, metabolomics and physiological analyses for dissecting the toxic effects of halosulfuron-methyl on soybean seedlings (*Glycine max* merr.). *Plant Physiol. Biochem.* **2020**, *157*: p, 303-315.
